# A Case of HIV-Associated Primary Central Nervous System Lymphoma Refractory to Methotrexate-Based Systemic Therapy and Immune Reconstitution

**DOI:** 10.7759/cureus.80273

**Published:** 2025-03-08

**Authors:** Joseph M Cleveland, Hayley A Cleveland, Erin Chamberlain, Jason Sinclair, Nam Ku, Levanto Schachter, Brian A Di Carlo

**Affiliations:** 1 Hematology and Medical Oncology, University of California, Los Angeles, San Luis Obispo, USA; 2 Infectious Diseases, University of California, Los Angeles, San Luis Obispo, USA; 3 Pathology, University of California, Los Angeles, Los Angeles, USA

**Keywords:** cmv encephalitis, cns lymphoma, hiv aids, hiv-associated lymphoma, immune reconstitution

## Abstract

Primary central nervous system lymphoma (PCNSL) exists as an HIV-associated malignancy, with its etiology driven primarily by Epstein-Barr virus (EBV) in the setting of absence of EBV-specific CD4+ T cell function, with CD4 counts averaging <50 cells/microliter. Before the development of antiretroviral therapy (ART), the treatment of HIV-associated PCNSL was primarily whole brain radiation therapy, which was fraught with long-term cognitive dysfunction. The availability of ART made the treatment of HIV-associated PCNSL possible to treat with curative intent. Here, we discuss a complex case of HIV-associated PCNSCL in a young male patient, wherein immune reconstitution combined with lymphoma-directed systemic therapy was ineffective in eradicating malignancy.

## Introduction

Fundamentally, immune reconstitution with normalization of the CD4 count has been considered the foundation of treatment for HIV-associated primary central nervous system lymphoma (PCNSL) [1-3]. High-dose methotrexate with leucovorin rescue and rituximab has been used in combination with antiretroviral therapy (ART) to increase responses and overall survival in this patient population [4]. The reason for the effectiveness of ART in HIV-associated PCNSL is due primarily to the concept that the pathophysiology of this disease is due to immune dysregulation (T-cell immunity) in the setting of HIV, leading to lack of control of viruses such as Epstein-Barr virus (EBV), which is the specific culprit virus in the majority of HIV-associated non-Hodgkin lymphomas, as in the case of PCNSL. There are hypotheses that EBV and HIV co-infection may lead to the proliferation of B-cell clones that may harbor mutations that may lead to the development of lymphoma. ART is deemed to be an effective modality of therapy given that it decreases the burden of HIV viral load, thus allowing augmentation of CD4 T-cell immunity and ultimately enhanced control of viruses such as EBV and subsequent suppression of B-cell clonal proliferation. Although treatment with the goal of cure provides significant hope for these patients, they remain at high risk for infectious complications in the setting of profound immunosuppression. Here, we present a case of a young man with newly diagnosed AIDS with PCNSL, who experienced a challenging course despite immune reconstitution. This case is particularly insightful, given that a clinical exam, imaging, and laboratory data that seemingly portend progression of malignancy may instead have a presentation that mirrors that of infection of an immunocompromised host. This case warrants attention for all clinicians to carefully consider infectious etiologies in all patients with PCNSL who may have objective concerns for progression.

## Case presentation

**Figure 1:**
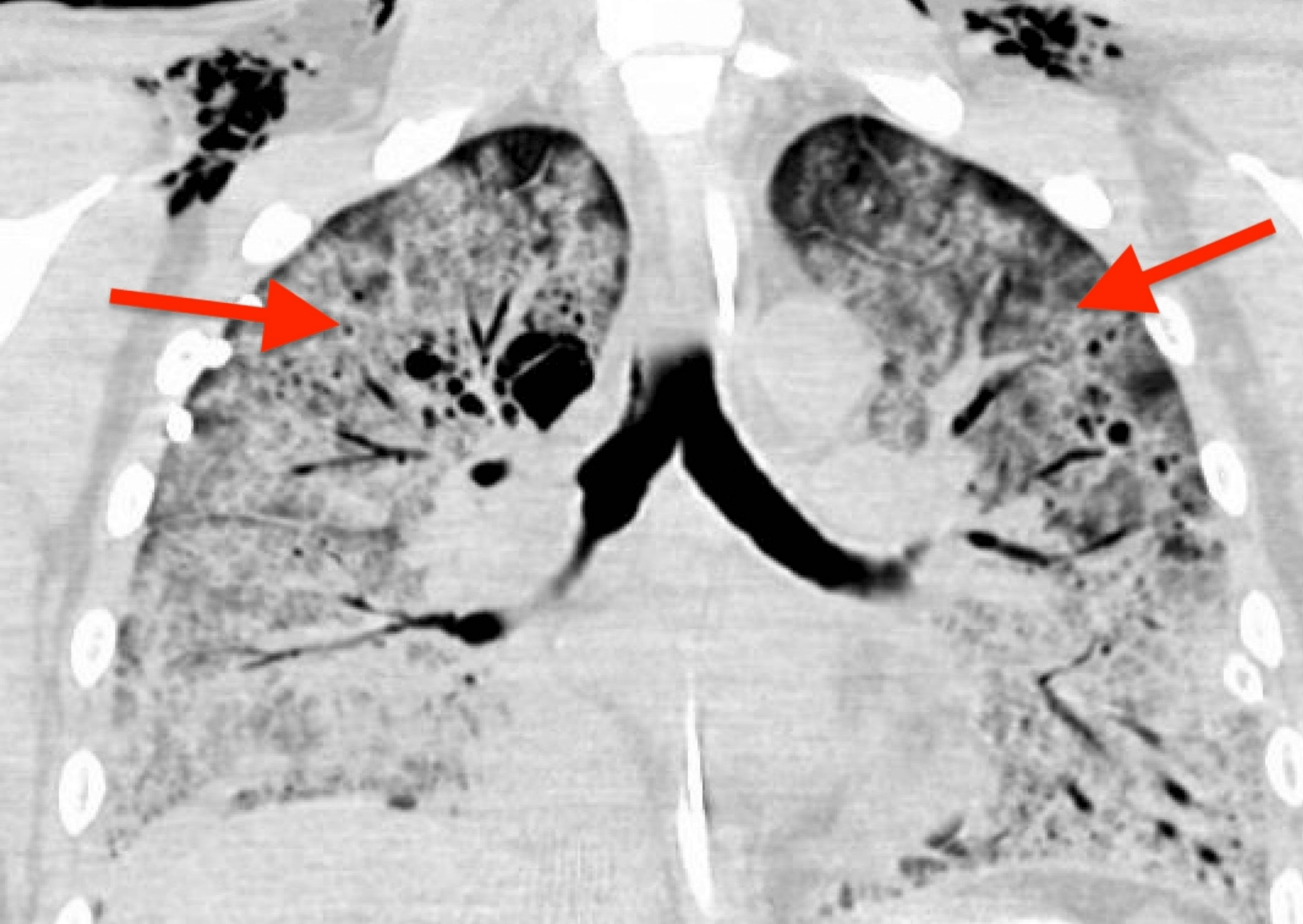
Coronal CT of the chest with contrast demonstrating bilateral ground glass opacities.

A 35-year-old previously healthy man presented to a local emergency room with dyspnea and fatigue, which appeared to be a subacute progression of symptoms encompassing a four-month decline in functional status manifested by night sweats, weight loss, and malaise. He was found to be profoundly hypoxic with oxygen saturation at 70%, febrile with a temperature of 101° Fahrenheit, tachycardic with a heart rate of 112 beats per minute, and hypotensive with a blood pressure of 80/56 mm Hg. He was transferred to the ICU and intubated. CT of the chest identified bilateral ground glass opacities, and bronchoscopy with lavage confirmed pneumocystis pneumonia (Figure 1).

His initial labs on presentation are reported below in Table 1. The laboratory data were significant for neutropenia, CD4 lymphopenia, and elevated HIV viral load by quantitative polymerase chain reaction (PCR).

**Table 1:** Initial laboratory values. HIV: human immunodeficiency virus; PCR: polymerase chain reaction; CD4: cluster of differentiation factor 4.

Lab	Value	Normal range
White blood cell count	1,700 cells/microliter	4,160-9,950 cells/microliter
Absolute neutrophil count	700 cells/microliter	1,800-6,900 cells/microliter
Hemoglobin	12.7 grams/deciliter	13.5-17.1 grams/deciliter
Mean corpuscular volume (MCV)	99.5 femtoliters	79.3-98.6 femtoliters
Platelet count	287,000 cells/microliter	143,000-398,000 cells/microliter
CD4 count	27 cells/cubic millimeter	441-2156 cells/cubic millimeter
CD4 T cell subset	4%	28-63%
HIV-1/2 antigen/antibody screen	Reactive	Nonreactive
HIV viral load by quantitative PCR	33,700 cells/milliliter	Not detected

**Figure 2:**
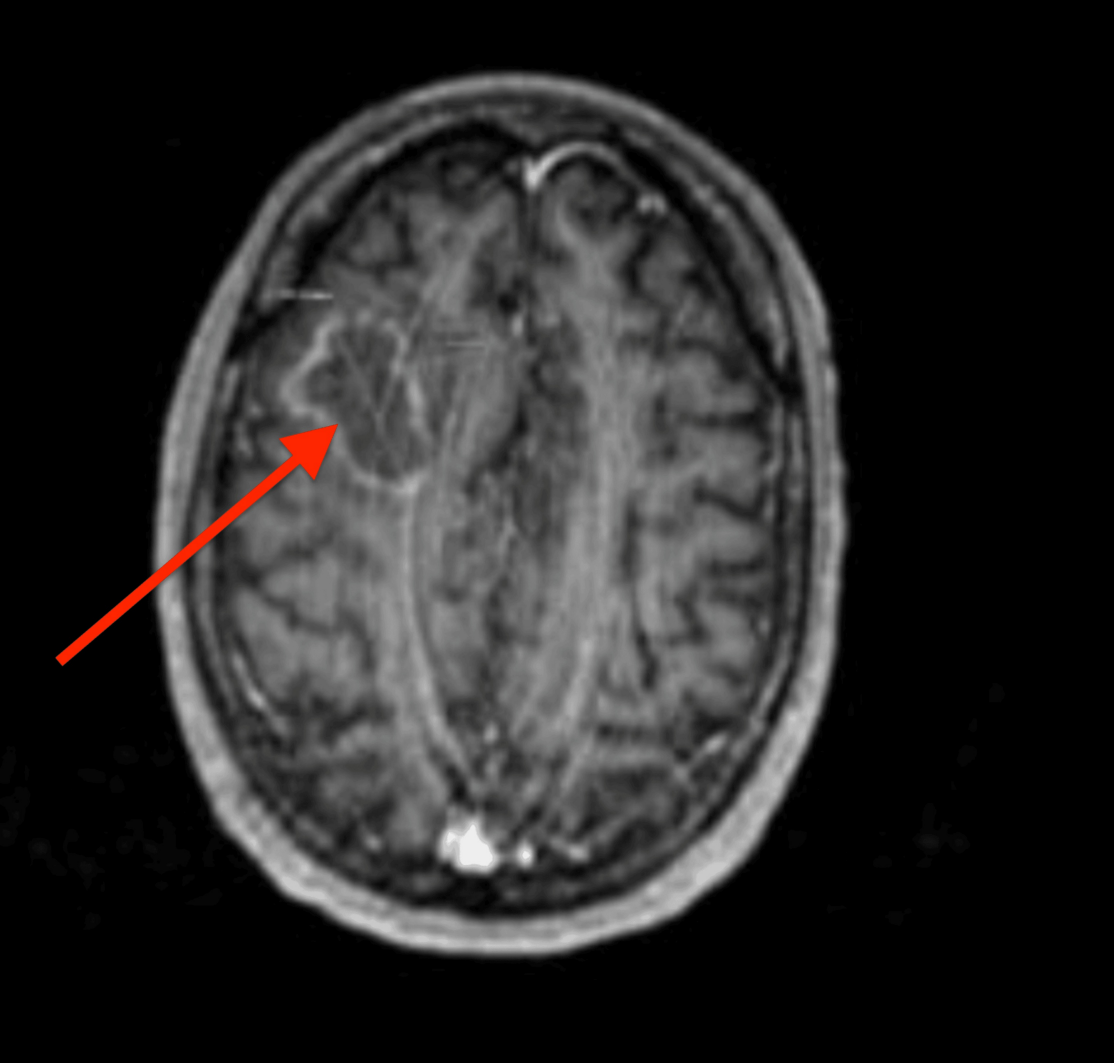
Right frontal lobe lesion.

**Figure 3:**
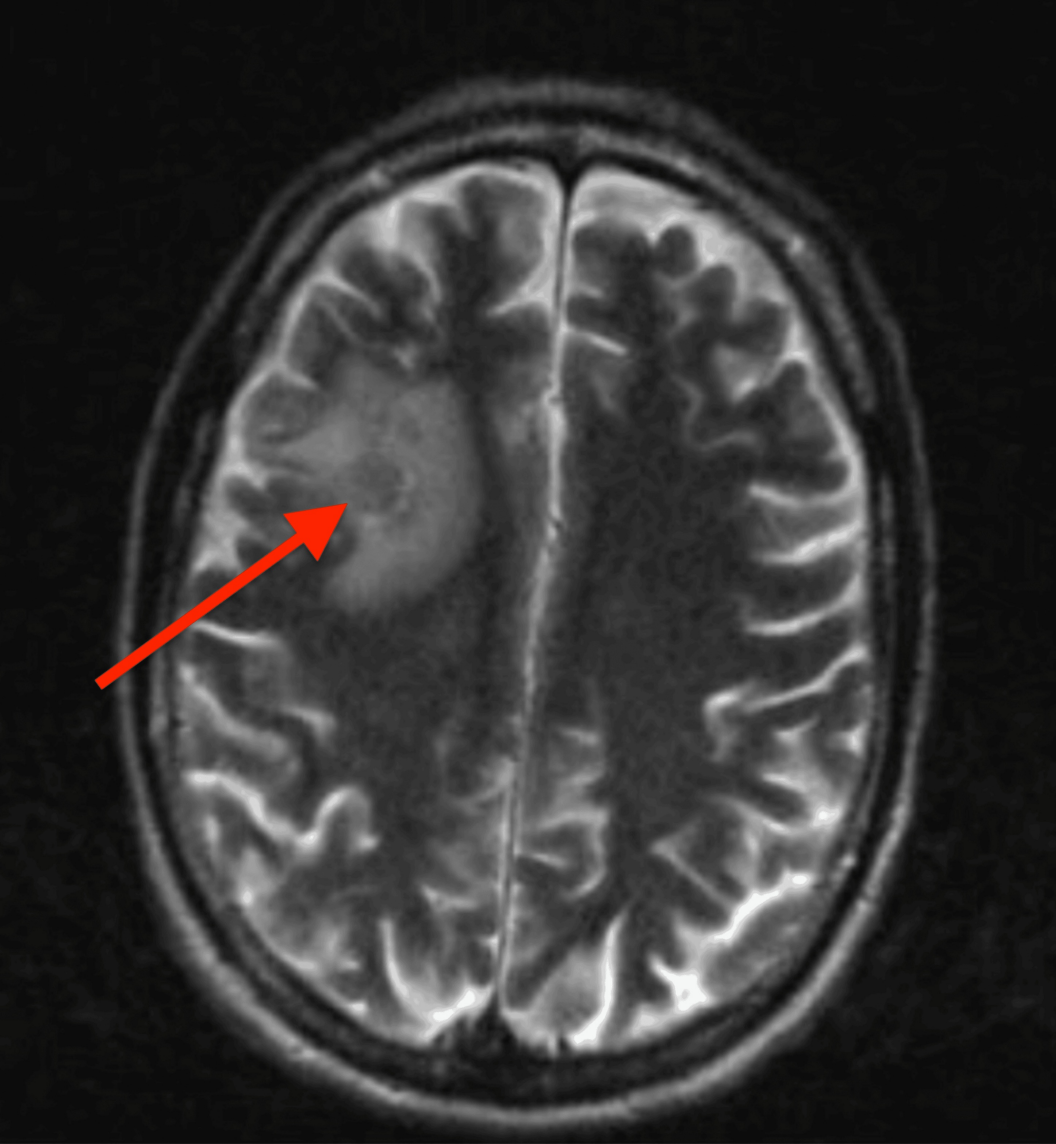
Vasogenic edema in the right frontal lobe.

Subsequently, brain imaging (MRI with contrast) was performed, given new onset seizures, and he was found to have a peripherally enhancing solid mass in the right frontal lobe, concerning for malignancy (Figures 2, 3).

**Figure 4:**
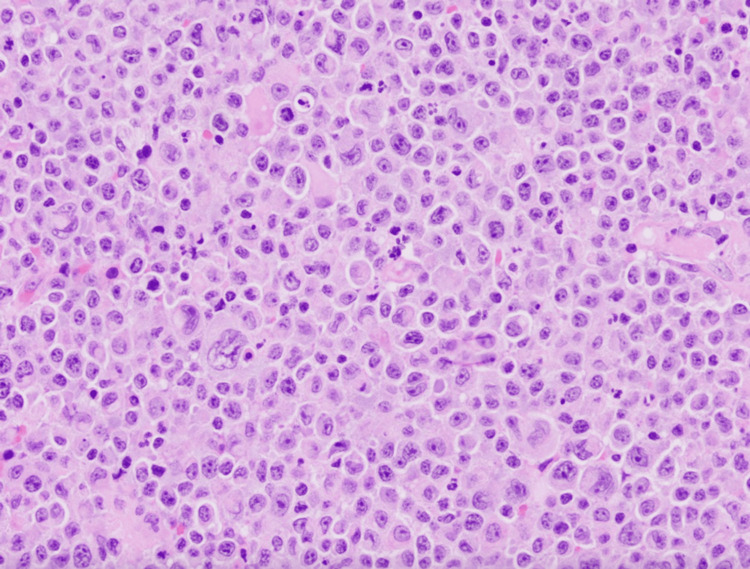
Hematoxylin and eosin (200x magnification) slide revealing diffuse effacement of lymph node architecture with large atypical lymphocytes with prominent nucleoli. Courtesy of Jason Sinclair, MD and Fausto Rodriguez, MD, Dept. of Neuropathology, University of California, Los Angeles (UCLA).

**Figure 5:**
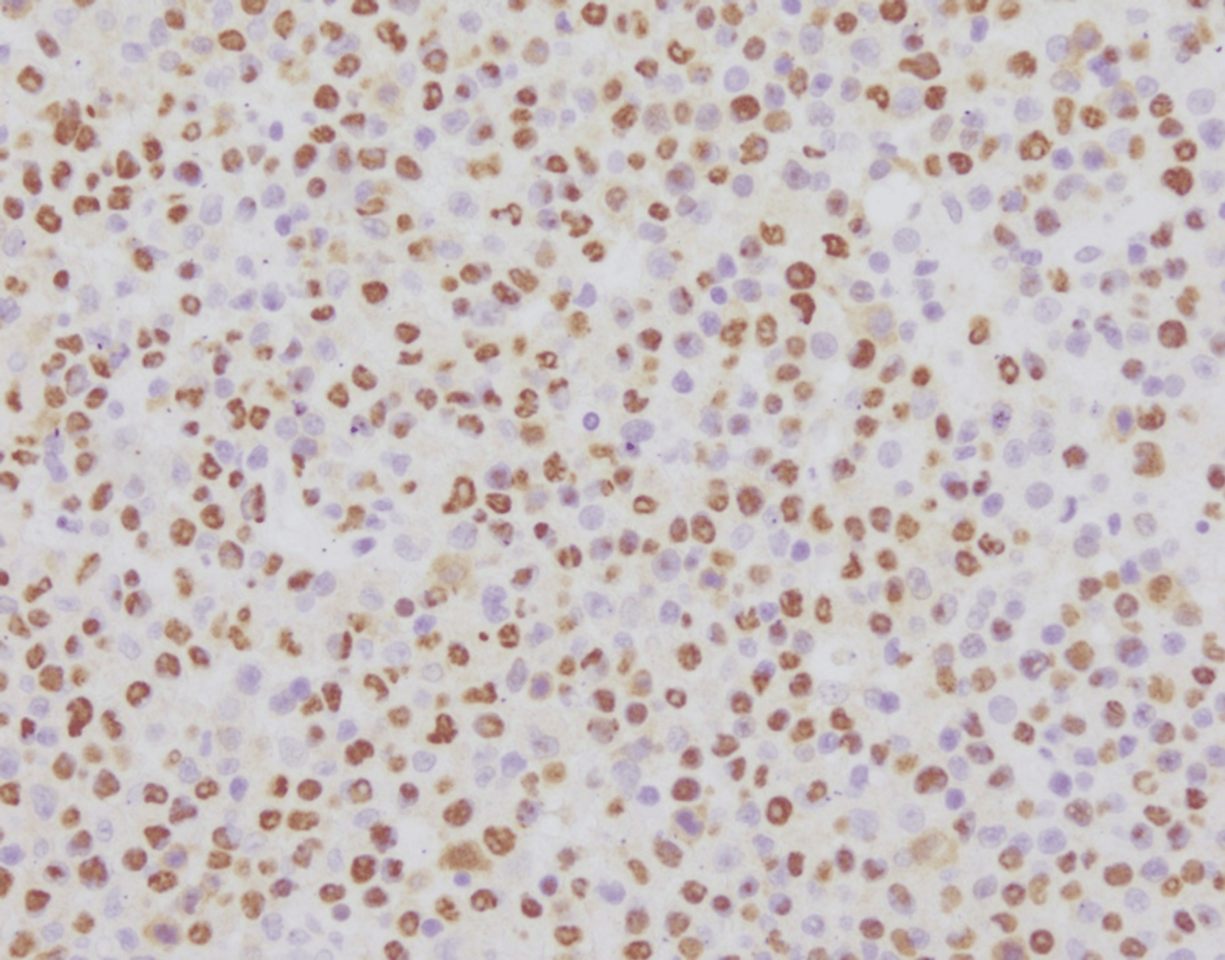
Neoplastic lymphocytes stain positive for Epstein-Barr virus-encoded small RNA (EBER) (200x magnification). Courtesy of Jason Sinclair, MD and Fausto Rodriguez, MD, Dept. of Neuropathology, University of California, Los Angeles (UCLA).

**Figure 6:**
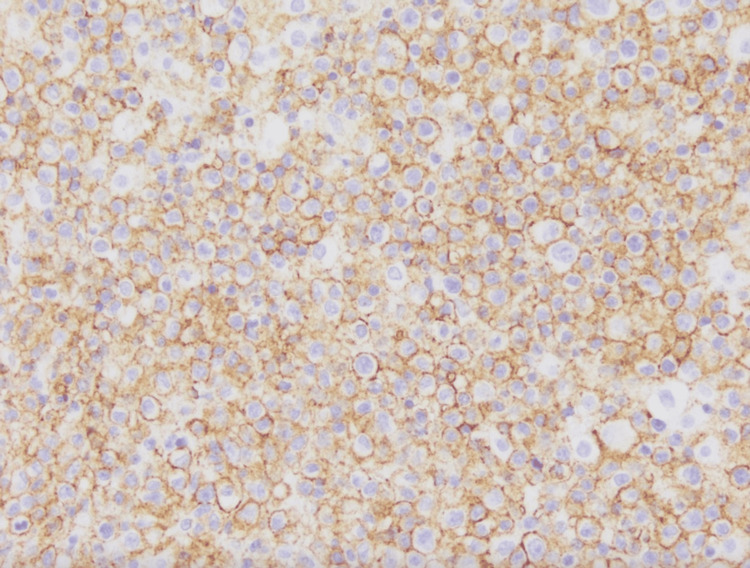
Neoplastic lymphocytes stain positive for CD20 (200x magnification). Courtesy of Jason Sinclair, MD and Fausto Rodriguez, MD, Dept. of Neuropathology, University of California, Los Angeles (UCLA).

The patient was initiated on treatment for pneumocystis pneumonia and transferred to a tertiary care facility for cannulation for venovenous (VV) extracorporeal membrane oxygenation (ECMO), given bilateral pneumothoraces that subsequently developed, as well as for urgent neurosurgical intervention. Once the patient was stabilized on VV ECMO, a right frontal craniotomy with biopsy was performed, confirming EBV-positive diffuse large B cell lymphoma (DLBCL), consistent with primary central nervous system (CNS) lymphoma in the setting of HIV (Figures 4-6).

**Figure 7:**
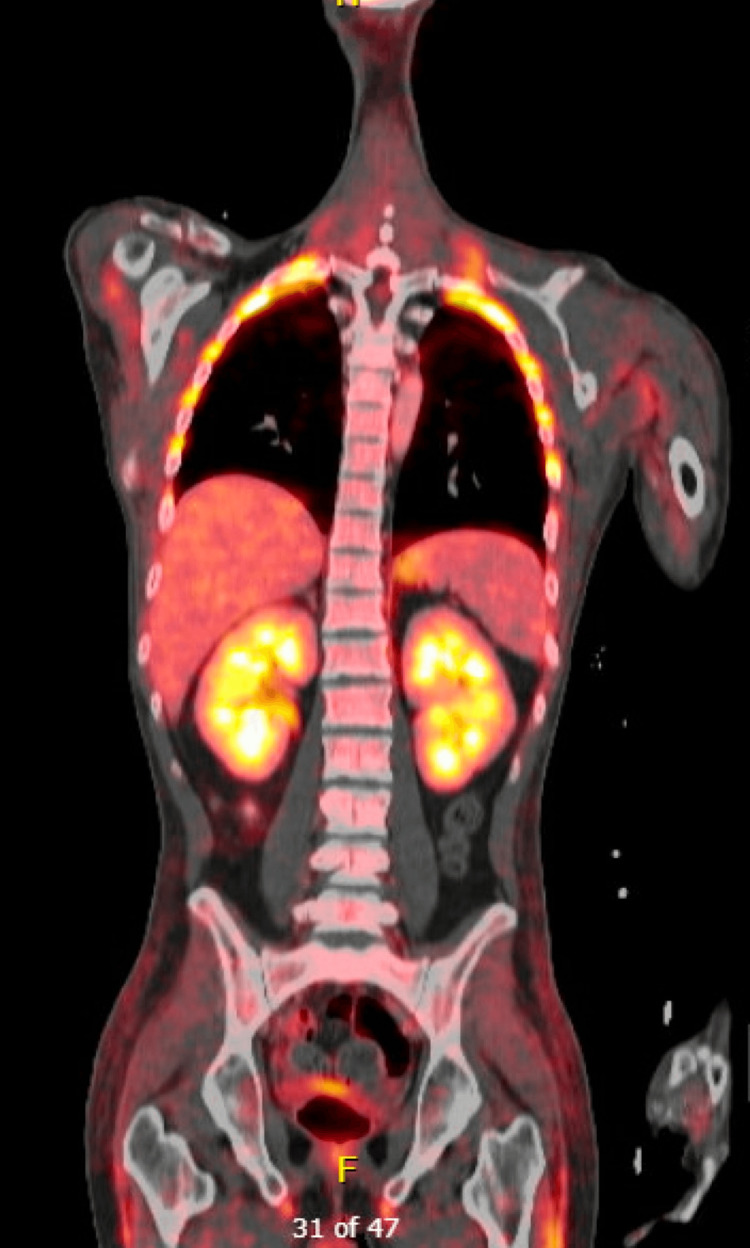
Whole body PET/CT negative for extracranial lymphoma.

Fluorodeoxyglucose (FDG)-PET/CT was negative for other evidence of systemic lymphoma (Figure 7).

**Figure 8:**
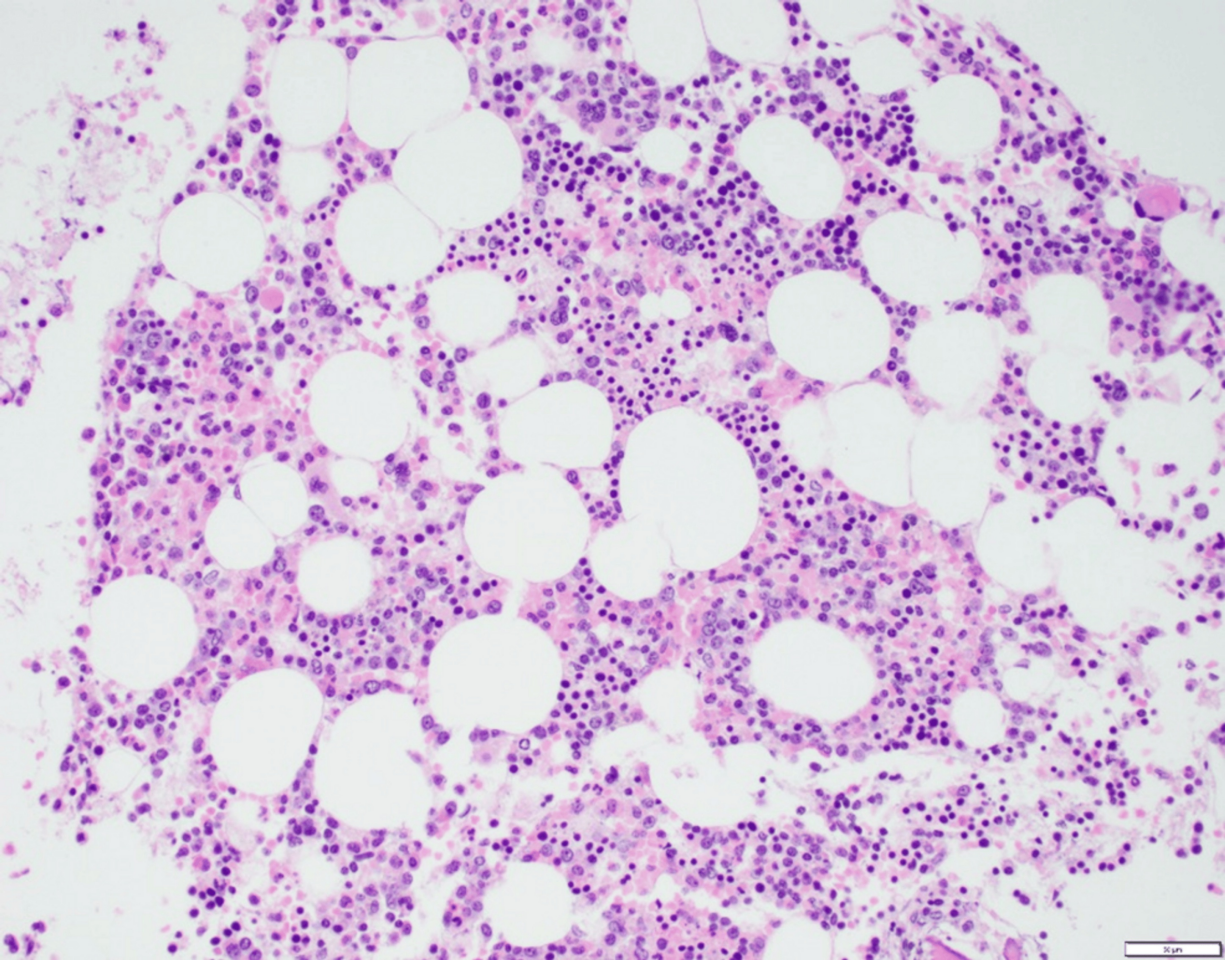
Hematoxylin and eosin (200X magnification) bone marrow biopsy slide showing mildly hypocellular marrow with no atypical large lymphoid infiltrate. Courtesy of Nam Ku, MD, Dept. Hematologic Pathology, University of California, Los Angeles (UCLA).

**Figure 9:**
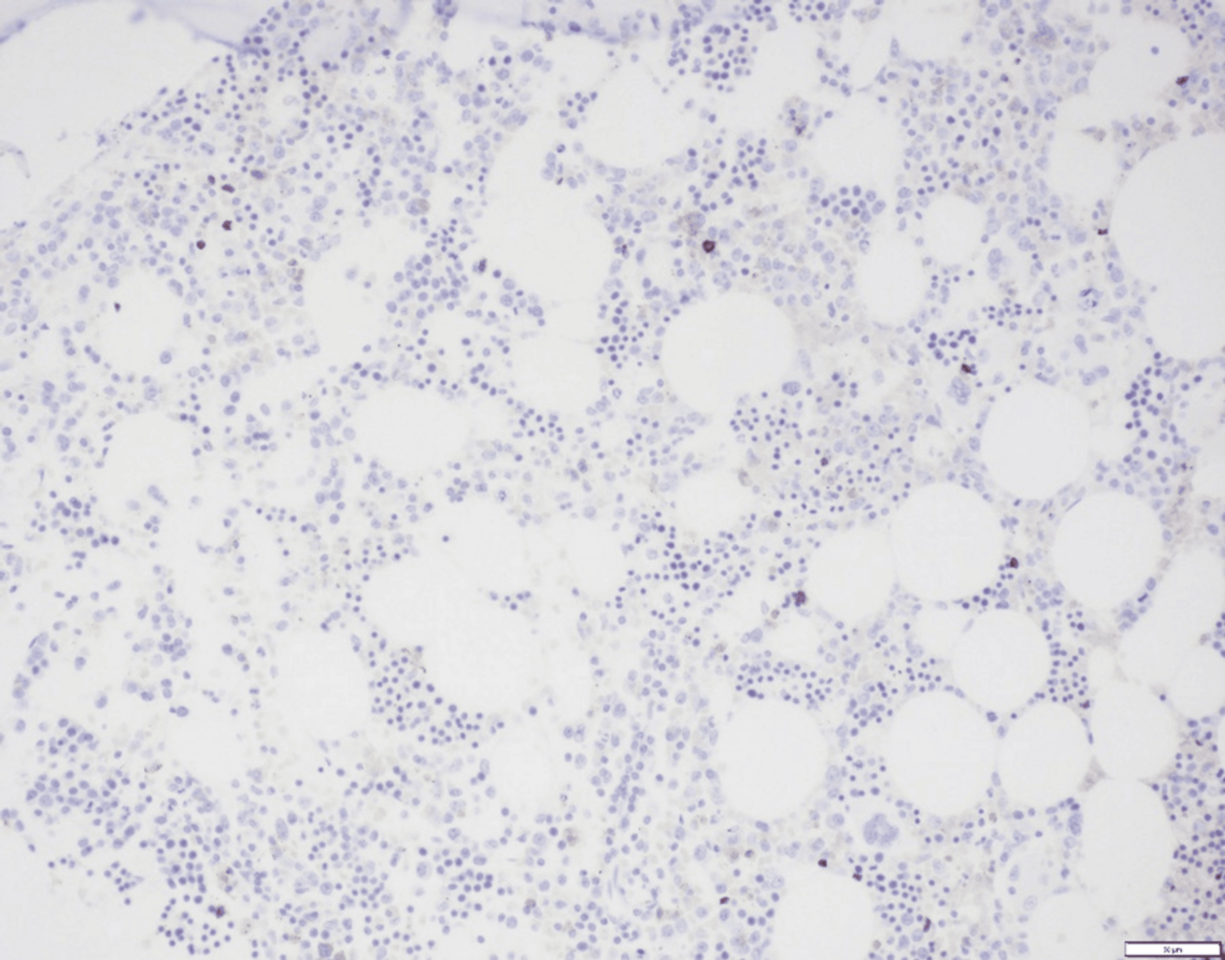
CD20 immunohistochemistry (200x magnification) slide of bone marrow highlighting only rare small B cells. Courtesy of Nam Ku, MD, Dept. of Hematologic Pathology, University of California, Los Angeles (UCLA).

Bone marrow biopsy was then obtained and was also negative for involvement with DLBCL (Figures 8, 9).

Cerebrospinal fluid (CSF) by lumbar puncture was negative for malignant B-cell population by flow cytometry or cytology.

The patient was then initiated on treatment with steroids and subsequently underwent four initial cycles of high-dose methotrexate and rituximab. He was simultaneously started on ART with bictegravir/emtricitabine/tenofovir alafenamide. His CD4 count, CD4 T-cell subset, and HIV viral load by quantitative PCR all improved (Table 2).

**Table 2:** Repeat laboratory values after initiating antiretroviral therapy. HIV: human immunodeficiency virus; PCR: polymerase chain reaction; CD4: cluster of differentiation factor 4.

Lab	Value	Normal range
CD4 T cell subset	11%	28-63%
HIV-1/2 antigen/antibody screen	Reactive	Nonreactive
HIV viral load by quantitative PCR	177 cells/milliliter	Not detected

**Figure 10:**
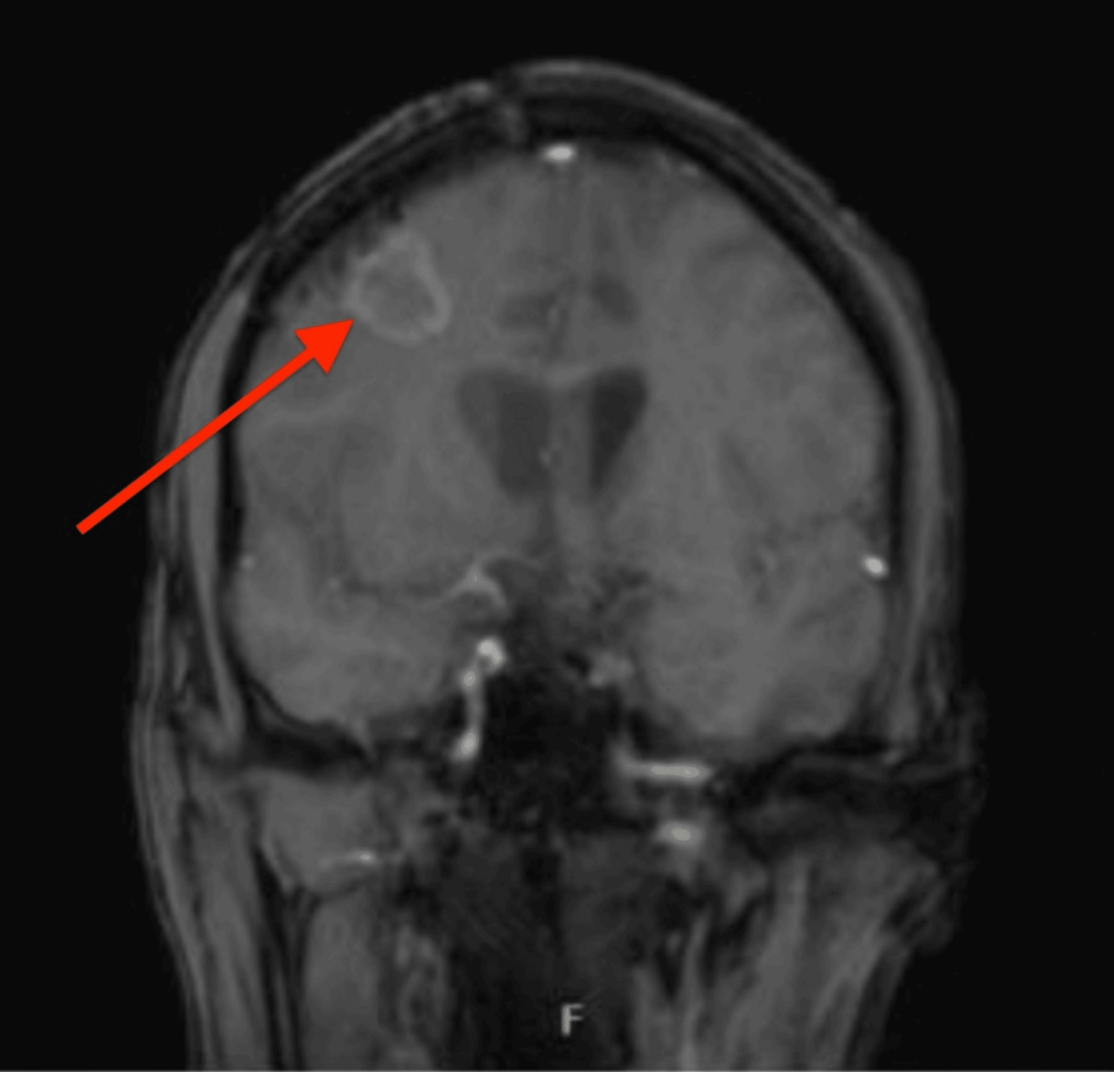
Coronal image of enhancing right frontal lobe lesion resection cavity.

**Figure 11:**
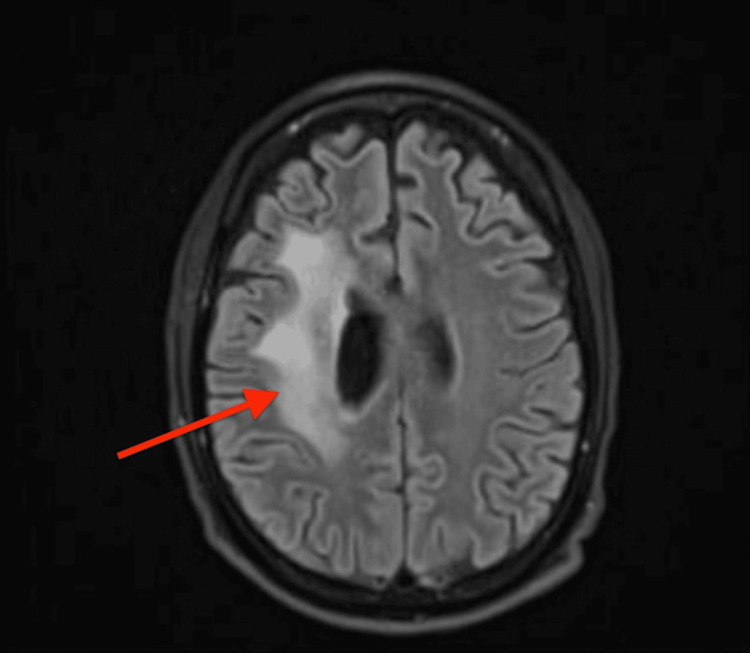
Transverse image showing leptomeningeal enhancement and edema extending to the right corona radiata.

A subsequent brain MRI showed stable disease. During the course of treatment, he developed cytomegalovirus (CMV) viremia and CMV retinitis. He was treated with oral and intraretinal valganciclovir. His CSF was positive for CMV, but the patient did not have clinical evidence of encephalitis. The patient went on to receive two additional cycles of high-dose methotrexate and rituximab. His brain MRI post six cycles unfortunately showed leptomeningeal enhancement at the boundary of the right frontal lobe resection cavity with extension to the right corona radiata, concerning for recurrence of CNS lymphoma per radiology (Figures 10, 11).

The patient subsequently developed severe hyponatremia and was experiencing recurrent falls, profound fatigue, and altered mental status (Table 3). His blood counts, which had previously normalized after initial systemic therapy and ART, now worsened with evidence of pancytopenia (Table 3). The patient was readmitted to the hospital and was found to have a CD4 count that had improved (though this was obtained after steroids were given, which may have caused a falsely elevated lab result). Subsequent CD4 counts after steroids were held revealed a value that was severely diminished. HIV viral load by quantitative PCR was significantly improved.

**Table 3:** Laboratory values during subsequent hospitalization. HIV: human immunodeficiency virus; PCR: polymerase chain reaction; CD4: cluster of differentiation factor 4.

Lab	Value	Normal range
Sodium	119 millimole/liter	135-146 millimole/liter
White blood cell count	1,000 cells/microliter	4,160-9,950 cells/microliter
Absolute neutrophil count	400 cells/microliter	1,800-6,900 cells/microliter
Hemoglobin	7 grams/deciliter	13.5-17.1 grams/deciliter
Platelet count	100,000 cells/microliter	143,000-398,000 cells/microliter
CD4 count (prior to steroids)	216 cells/cubic millimeter	441-2156 cells/cubic millimeter
CD4 count (after steroids held)	6 cells/cubic millimeter	441-2156 cells/cubic millimeter
CD4 T cell subset	2%	28-63%
HIV-1/2 antigen/antibody screen	Reactive	Nonreactive
HIV viral load by quantitative PCR	<20 cells/milliliter	Not detected

Per the family, the patient was reportedly consistent with taking his ART. Given the lack of improvement in CD4 reconstitution, rather than assumed pure progression of malignancy, infection was included on the differential diagnosis. The patient underwent repeat infectious workup, which confirmed CMV qualitative positivity in the CSF (Table 4).

**Table 4:** CMV qualitative PCR of cerebrospinal fluid. PCR: polymerase chain reaction; CMV: cytomegalovirus.

Lab	Value	Normal range
CMV qualitative PCR	Detected	Not detected

**Figure 12:**
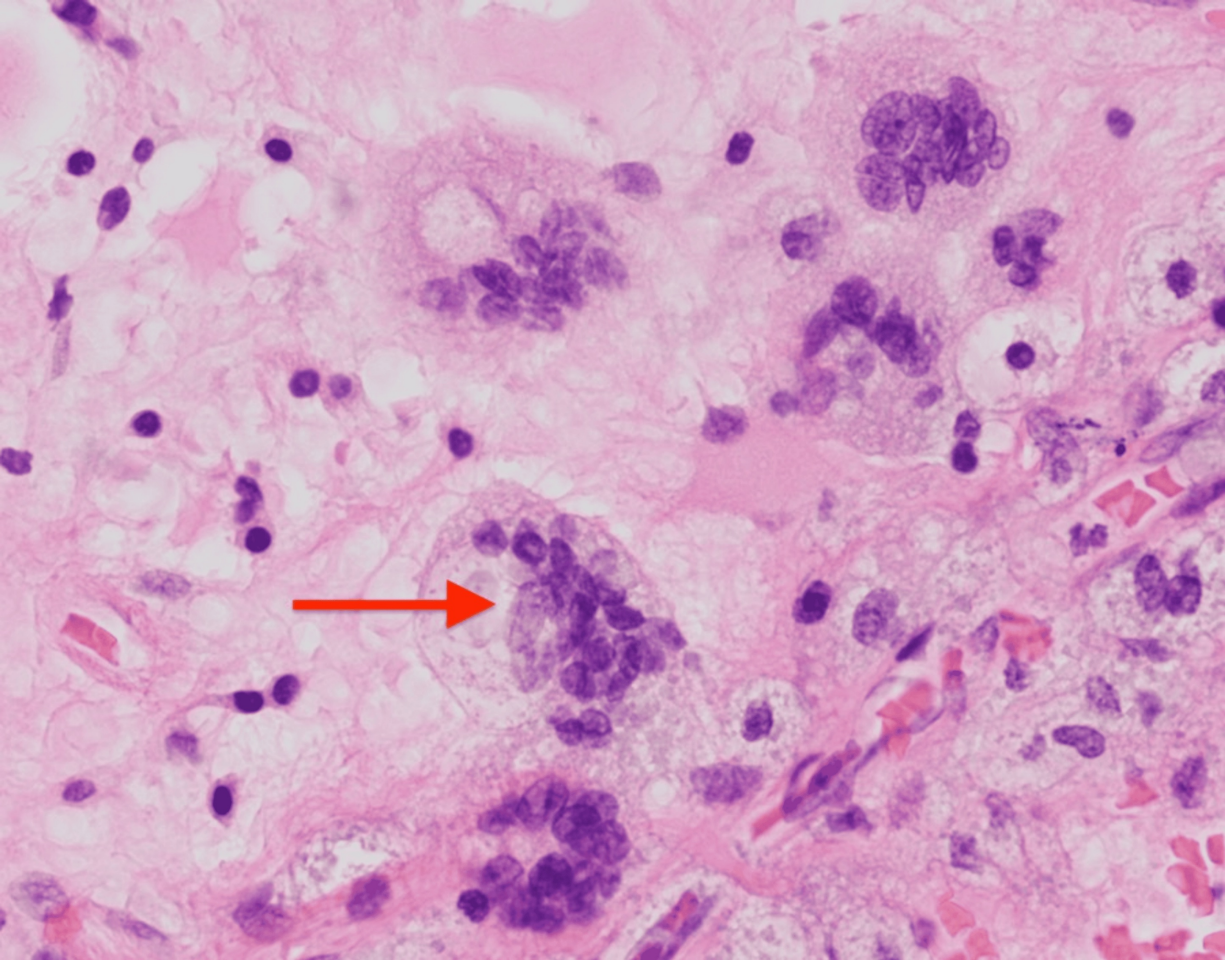
Hematoxylin and eosin (400x magnification) brain biopsy revealing multi-nucleated giant cells.

The patient was started on intravenous (IV) ganciclovir. A brain biopsy ruled out CNS lymphoma but instead revealed CMV encephalitis (neuroglial tissue with necrosis, chronic inflammation, and multinucleated giant cells) (Figure 12).

CMV qualitative PCR of the brain biopsy was also positive (Table 5).

**Table 5:** CMV qualitative PCR of the brain biopsy. PCR: polymerase chain reaction; CMV: cytomegalovirus.

Lab	Value	Normal range
CMV qualitative PCR	Detected	Not detected

Despite treatment, the patient had persistent deterioration with progressive somnolence and subsequently transitioned to comfort care and died shortly thereafter.

## Discussion

HIV-associated PCNSL is an AIDS-defining malignancy that is inherently linked to EBV in the setting of CD4 lymphopenia [2]. Common presenting symptoms include altered mental status, seizures, focal neurological deficits, and poor oral intake.

HIV-PCNSL is often identified on imaging to present as a well-circumscribed hyperintense mass on brain MRI, favoring a supratentorial location in the cerebral hemispheres [1].

The differential diagnosis for brain lesions in a patient with CD4 lymphopenia (CD4 < 200 cells/microL) includes toxoplasmosis, CMV encephalitis, fungal abscesses, tuberculosis, or progressive multifocal leukoencephalopathy (PML) [4].

The accurate diagnosis of PCNSL requires stereotactic biopsy, and steroids should be reserved until after diagnosis is obtained if clinical status allows [5]. CSF flow cytometry can also be utilized, though a negative result does not rule out the diagnosis [6].

This case highlights the importance of prompt diagnosis and treatment of infectious diseases in patients with profound immunodeficiency in the setting of HIV-associated PCNSL [7,8]. Additionally, it underscores that not all patients treated with ART with lymphoma-directed therapy will experience proper immune reconstitution or disease control. Possible contributions to lack of immune reconstitution could be inherently related to the fact that different HIV subtypes may develop resistance to ART, thus leading to ineffective control of HIV-associated PNCSL. Similarly, although this patient was on CMV prophylaxis, he still subsequently developed CMV encephalitis, which ultimately led to his clinical deterioration and also raised concerns for the resistance of CMV to antiviral therapy. This case is not isolated as the literature does suggest that lack of response to ART and systemic therapy is often observed in patients with inadequate HIV viral load suppression and CD4 augmentation [9]. Clinicians treating patients with HIV-associated PCNSL must remain cognizant throughout the course of treatment for continued CD4 augmentation with close lab monitoring of CD4 T-cell subsets and frequent clinical exams to assess for occult development of infection. Furthermore, even despite aggressive lymphoma-directed systemic therapy, without successful immune reconstitution, mortality from both infection and malignancy within an immunocompromised host remains significant [10,11].

## Conclusions

The foundation of treatment for patients with HIV-associated PCNSL is ART in combination with high-dose methotrexate with leucovorin rescue, with or without rituximab. High-dose chemotherapy with autologous stem cell rescue is not commonly used in this population of patients, given that the profound immunosuppression in the setting of autologous stem cell transplant can potentially worsen HIV infection or any associated opportunistic infection. CMV, among other infectious etiologies such as toxoplasmosis, pneumocystis pneumonia, and tuberculosis, is a potential opportunistic infection that can arise in the setting of HIV and can complicate the treatment of HIV-PCNSL, even in the setting of lymphoma-directed systemic therapy. Future research directions include analysis of HIV subtypes that are prone to resistance to ART as this may better predict patients who may have a higher risk of nonresponse to immune reconstitution and, ultimately, progression of malignancy. Immune reconstitution remains the core of treatment, and if it is not achieved, then opportunistic infections may thwart treatment of PCNSL. Although treatment with ART and high-dose methotrexate-based regimens can potentially offer a cure, opportunistic infections frequently are responsible for the significant morbidity and mortality in this patient population, emphasizing the importance of prompt recognition and treatment of these deadly comorbidities.
